# A Rare Case of Congenital Buccal Lipoblastoma in a Term Neonate

**DOI:** 10.7759/cureus.75461

**Published:** 2024-12-10

**Authors:** Vonita Chawla, Indirapriya Avulakunta, Jeffrey A Dorrity, Kandi A Stallings-Archer, Adam Johnson, Sarah M Perez

**Affiliations:** 1 Pediatrics/Neonatology, University of Arkansas for Medical Sciences, Little Rock, USA; 2 Otolaryngology, University of Arkansas for Medical Sciences, Little Rock, USA; 3 Pathology, University of Arkansas for Medical Sciences, Little Rock, USA

**Keywords:** benign oral mass, buccal mass, lipoblastoma, neonate, oral tumor

## Abstract

A lipoblastoma is a benign tumor of adipocytes originating from embryonic white fat and occurs in the pediatric population. Congenital lipoblastomas, however, are rare, and the incidence of these tumors in neonates is unknown. Due to their rare presentation, congenital oral lipoblastomas can, firstly, pose diagnostic challenges for the pediatrician and must be differentiated from the more commonly seen oral lesions in the newborn and other rare malignant growths. Secondly, these benign yet gradually enlarging tumors may result in obstructive and/or compressive symptoms, an important consideration for congenital tumors present in the facial, head, neck, and oral locations given impending airway compromise, dysphagia, and feeding difficulties associated with large growths. We report a rare case of congenital buccal lipoblastoma in a term female newborn presenting as a pedunculated mass arising from the left buccal mucosa. She underwent surgical excision, and histopathological analysis revealed the mass to be a lipoblastoma. Subsequently, the infant recovered well and has had no recurrence of the mass. Follow-up is recommended post-excision, given the risk of recurrence, which is higher with incomplete resection and lipoblastomatosis (a deep, infiltrative, ill-defined subtype of lipomatous tumors). The pleomorphic adenoma gene 1* (PLAG 1) *gene overexpression has been implicated in a majority of lipoblastomas and may aid in the diagnosis of atypical tumors. Oral lipoblastoma should be considered in the differential diagnoses for newborns presenting with a mass or growth in the oral cavity.

## Introduction

Lipoblastomas have been recognized in the literature as tumors arising from embryonic white fat, seen most often in children [1]. However, reports of congenital lipoblastomas presenting in the newborn period are scarce. Due to their infrequent occurrence in newborns, these tumor-like growths can prove to be diagnostic dilemmas for the neonatal provider, who may not be familiar with this unusual clinical finding. Newborns born with tumors within the oral cavity can show a varied presentation, from being completely asymptomatic to having severe, life-threatening symptoms due to airway obstruction and respiratory failure, adding to the complexity of medical decision-making [2]. Clinical presentation often depends upon the size and location of the lesion and, as such, requires a tailored therapeutic approach. Here, we present a rare case of congenital buccal lipoblastoma in a newborn.

## Case presentation

A female newborn was referred to our Neonatal Intensive Care Unit (NICU) for a buccal mass noted shortly after birth. The baby was born at a gestational age of 39 weeks and five days via emergent Caesarian section (C-section) due to fetal distress following spontaneous labor to a twenty-seven-year-old Gravida 2, Para 2, African American mother. Pregnancy was complicated by the presence of a short cervix. The mother received routine prenatal care and did not receive any medications during pregnancy other than prenatal supplements. The mother’s prenatal labs were unremarkable. There was no history of smoking, alcohol consumption, or any illicit substances during pregnancy. Spontaneous rupture of membranes occurred two hours prior to C-section. Meconium was present in the amniotic fluid, and at delivery, the umbilical cord was wrapped around the baby’s chest. The newborn was vigorous at delivery and weighed 3.16 kilograms. APGAR scores were 8 and 9 at 1 and 5 minutes, respectively. The baby received vitamin K, the hepatitis B vaccine, and erythromycin eye ointment at birth. At two hours of life, while being bottle-fed, the patient was noted to have an oral mass on her left buccal surface. The infant was made nothing by mouth (NPO) by the referring hospital upon discovery of the mass, and further attempts at oral feeding were not made. She was subsequently transferred to our facility for further evaluation.

**Figure 1:**
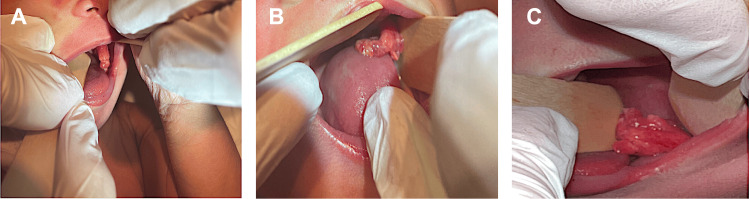
Images of buccal mass (Panel A, B, C): Images of fleshy-appearing left buccal mass with well-defined margins inside the oral cavity of a newborn.

Upon arrival, the neonate appeared to be active and in no respiratory distress. Her vital signs were within acceptable range for her age. Examination of her oral cavity revealed an approximately 2-centimeter pedunculated soft tissue mass originating from the left posterior buccal surface, with a 5-millimeter-long stalk (Figure 1, panels A, B, C).

The remainder of her physical exam was normal. Her laboratory tests, including a complete blood count, renal function panel, and capillary blood gas evaluation, were unremarkable (Table 1).

**Table 1:** Laboratory tests Screening lab tests obtained on admission

Laboratory test	Result	Reference range
Complete blood count		
Red blood cell count	5.5 million/µL	3.9 – 5.5 million/µL
Hemoglobin	21 g/dL	13.5 – 24 g/dL
Hematocrit	59.1%	42 – 60%
Mean corpuscular volume	106.7 fL	98 – 118 fL
Mean corpuscular hemoglobin	37.9 pg	31 – 38 pg
Mean corpuscular hemoglobin content	35.5 g/dl	30 – 36 g/dL
White blood cell count	19.58 x 10^3^/µL	9 – 30 x 10^3^/µL
Neutrophils	70.8%	66 – 87%
Lymphocytes	16.6%	20 – 37%
Monocytes	11.7%	4 – 12%
Eosinophils	0.2%	1 – 4%
Basophils	0.7%	0 – 1%
Platelet count	299 x 10^3^/µL	150 – 400 x 10^3^/µL
Mean platelet volume	9.8 fL	10 – 12 fL
Renal function panel		
Sodium	139	133 – 145 mmol/L
Potassium	4.3	3.2 – 6.3 mmol/L
Chloride	106	98 – 110 mmol/L
Carbon dioxide	19	18 – 27 mmol/L
Glucose	74	50 – 100 mg/dL
Calcium	10	6.9 – 10 mg/dL
Blood urea nitrogen	7	<5 – 19 mg/dL
Phosphorus	5.1	4.8 – 8.2 mg/dL
Albumin	4.1	2.6 – 3.6 g/dL
Creatinine	0.9	0.1 – 1.2 mg/dL
Arterial blood gas		
pH	7.4	7.32 – 7.49
pCO_2_	33	26 – 41 mm Hg
pO_2_	95	60 – 80 mm Hg
HCO_3_	22	16 – 24 mmol/L
Base deficit	4	0 – 2

The infant’s blood type was B-positive. Some initial differential diagnoses included mucosal cyst, ectopic thyroid tissue, and dental lamina cyst; however, the location and appearance of the patient’s mass did not match the typical presentation of these lesions.

**Figure 2:**
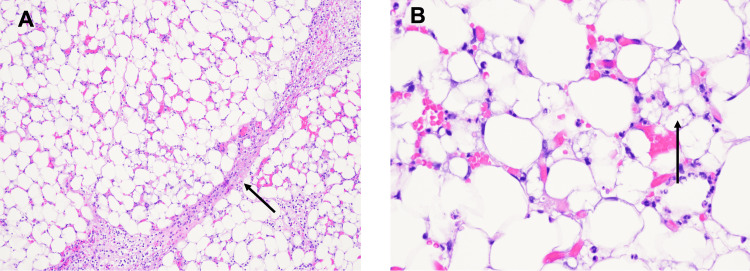
Histopathological images (Panel A): 100x magnification, hematoxylin and eosin (H&E) stain showing adipocytes separated by fibrous septum (arrow); (Panel B): 400x magnification, H&E stain showing adipocytes in variable stages of maturation, including multivacuolated lipoblasts (arrow).

Pediatric otolaryngology was consulted, and a complete excision of the mass was performed at the bedside with local anesthesia and no complications on the day of life (DOL) 2. Additional imaging was not deemed necessary due to the superficial, isolated, and well-encapsulated nature of the mass. The excised mass was sent for histopathologic analysis. Microscopically, the mass was composed of lobules of adipocytes separated by fibrous septae (Figure 2, panel A) with a background of marked, acute inflammation and necrosis. The adipocytes displayed variable stages of maturation, including multivacuolated lipoblasts (Figure 2, panel B). These morphologic findings were consistent with a diagnosis of lipoblastoma.

Post-excision, the infant was able to feed, void, and stool normally and was discharged home on DOL 4. Her newborn metabolic screens were within normal limits. She was followed as an outpatient by pediatric otolaryngology at three weeks and then again at four months of life. She showed no signs of recurrence of the lesion or development of new lesions, and her surgical site was healing well. The infant continues to grow appropriately and has no issues with feeding. 

## Discussion

Lipomatous tumors are rare soft tissue tumors (10%) of childhood [1]. The incidence of these lesions in the neonatal population is unknown. One such lesion is a lipoblastoma, which is benign in nature and presents as a well-circumscribed growth of embryonic white fat [3,4]. Primarily pediatric tumors, lipoblastomas are typically diagnosed within the first decade of life, although reports of congenital tumors are exceedingly rare. Similarly, liposarcomas (malignant adipose tissue sarcomas) are also rarely seen in neonates, and the exact incidence remains unknown. However, liposarcomas tend to affect pediatric patients in the second decade of life. Lipoblastomas tend to have a male predilection [5,6] and most commonly occur in the extremities or on the trunk [7] but can occur in other regions. 

Although benign, these tumors tend to progressively enlarge and depending upon the site of occurrence, can cause symptoms from compression of surrounding tissues. Consequently, facial, oral, and neck lesions pose unique clinical, diagnostic, and therapeutic challenges in the neonatal population. Neonatal patients may have airway obstruction or compression which can clinically present as respiratory distress, a clinical entity with a broad differential diagnosis in the newborn, and thus a high index of suspicion needs to be maintained to ensure timely evaluation and intervention. Wan MH et al in 2021, reported a lingual lipoblastoma in a newborn female that presented with respiratory distress and airway obstruction shortly after birth [2]. Additionally, neonates may present with feeding difficulties. For both symptomatic and asymptomatic neonates, interdisciplinary discussions regarding the timing of surgical intervention, the need for transfer to a facility with a higher level of care, and in-patient monitoring versus outpatient follow-up, are recommended by the authors. Lastly, the approach to a neonatal patient with lipoblastoma may be different from that of an older child with lipoblastoma. Older pediatric patients may present with additional symptoms, especially in the case of abdominal tumors. Consequently, the approach to surgery will also vary depending on the location and size of the tumor.

Congenital lipoblastomas should be differentiated from other oral lesions such as mucosal cysts, congenital epulides, ranulas, ectopic thyroid tissue, choristomas, hamartomas, and vascular lesions [8,9] in the newborn. A detailed physical exam is the first step, as the anatomical location of the mass can provide clues into underlying pathology. Lipoblastomas, which are generally encapsulated, must also be differentiated from other lipomatous growths, including lipoblastomatosis [1], which is less well-defined and tends to be infiltrative. This distinction is important as incomplete resection leads to recurrence in up to 25% of cases [10] due to the infiltrative nature of the lesion. Other differential diagnoses, albeit rare, include liposarcoma of varying degrees of differentiation, lipomas, atypical lipomatous tumors, and lipomatosis [1]. 

Histologically, a lipoblastoma consists of lobules of adipocytes in multiple stages of maturation and is separated by fibrous septae [11]. Abnormal pleomorphic adenoma gene 1 (PLAG 1) rearrangement (due to alteration in chromosome 8) and overexpression of PLAG 1 protein are implicated in up to 70% of lipoblastomas [11], and testing may be warranted if tumors are grossly or histologically atypical [12]. A follow-up period of at least five years is recommended [13], although some authors feel this should be longer based on institutional data [14,15].

## Conclusions

Congenital oral lipoblastomas are rare, and depending on size and location, these can present as a painless mass or swelling or be symptomatic at birth, potentially resulting in airway compromise and feeding difficulties; as such, a high index of suspicion must be maintained. Lesions present at birth may not get diagnosed until later when they are significantly larger. Pathology is diagnostic; however, cytogenetic analyses for the aberrant PLAG 1 gene may be helpful in diagnostically challenging lesions. The prognosis is excellent after complete resection. However, follow-up is recommended for a minimum of five years due to the risk of recurrence.
